# Supplementary figures and images for: Tracking SARS-CoV-2 variants through pandemic waves using RT-PCR testing in low-resource settings
Source: PLOS Glob Public Health. 2023 Jun 1;3(6):e0001896. doi: 10.1371/journal.pgph.0001896 (PMC10234525; doi:10.1371/journal.pgph.0001896)

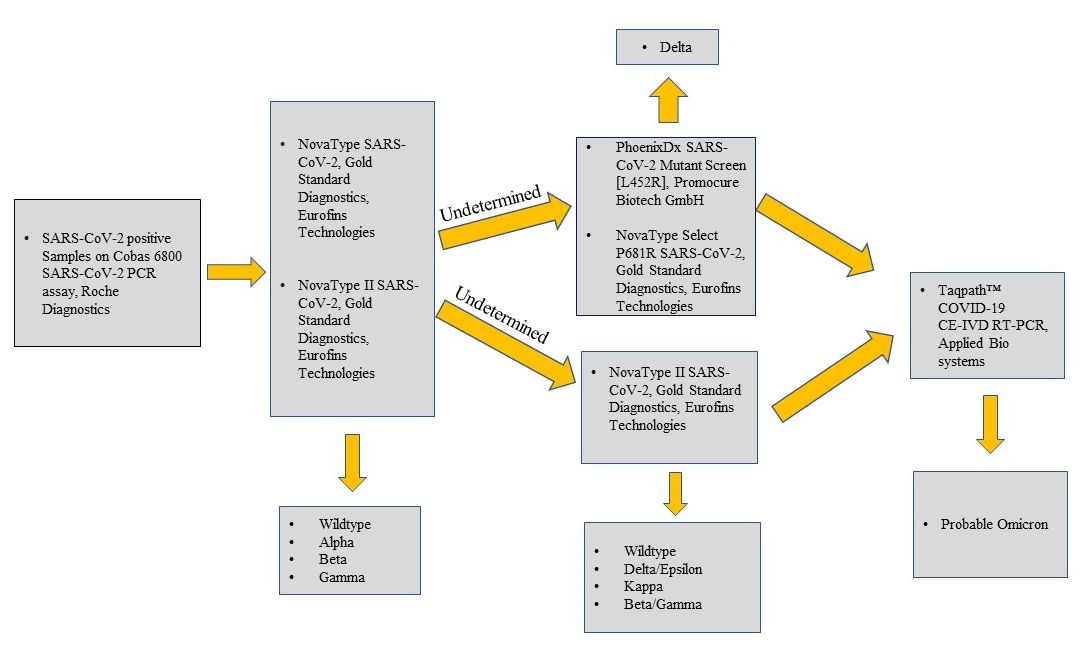

Supplement: S1 Fig — The GSD NovaType SARS-CoV-2 assay was utilized to screen for Alpha and Beta variants. The test differentiates Alpha variant (N501Y and A570D) from Beta variant (N501Y). Subsequently, a newer version of the assay, GSD NovaType II SARS-CoV-2 assay was utilized to identify Alpha (N501Y), Gamma (E484K, N501Y) and Beta (N501Y, E484K, K417N) variants. Delta variants were also screened in parallel by using the assays PhoenixDx SARS-CoV-2 Mutant Screen [L452R] and NovaType Select P681R SARS-CoV-2 an assay that identified Delta variants by the L452R and P681R mutations. The NovaType III SARS-CoV-2 assay identified key lineage mutations for; Delta/Epsilon (E484, L452R), Kappa (E484Q, L452R) and Beta/Gamma (E484K) variants. The Omicron variant (BA.1) had been identified by utilizing TaqPath COVID‑19 CE‑IVD RT‑PCR assay, omicron variants resulted in a S- gene Target Failure (SGTF). (TIF) [file pgph.0001896.s001.tif]

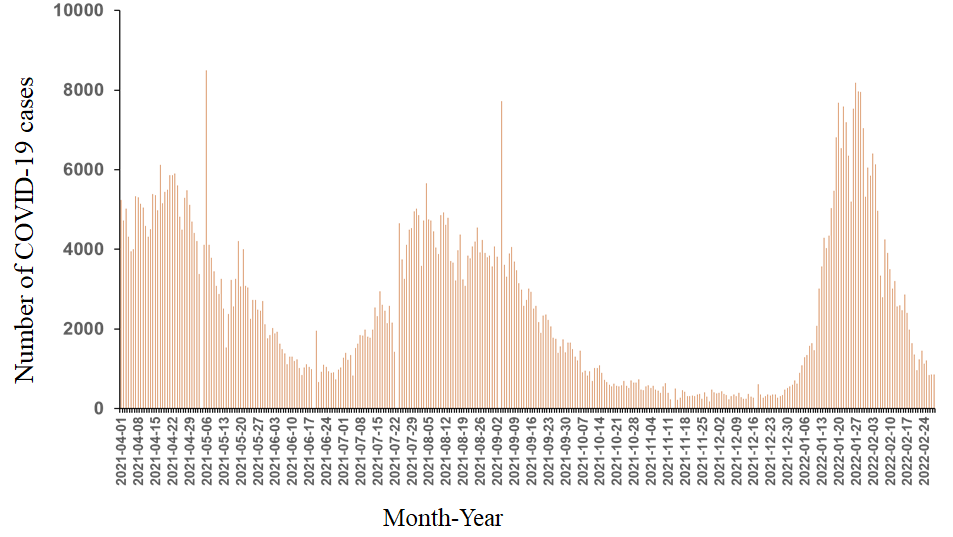

Supplement: S2 Fig — Data presented is for the COVID-19 positive cases in Pakistan between months April 2021 till February 2022. Source, John Hopkins, Corona Research Center https://coronavirus.jhu.edu/region/pakistan. (TIF) [file pgph.0001896.s002.tif]

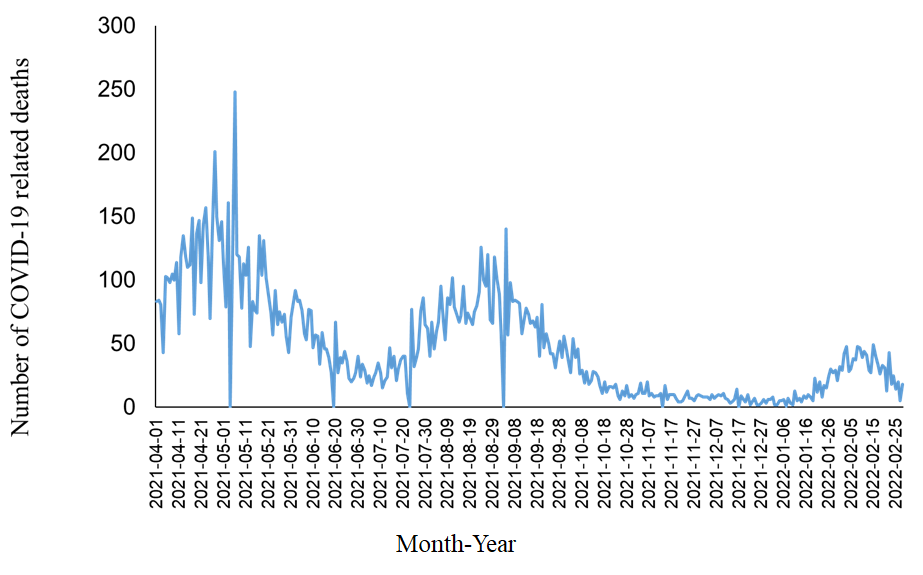

Supplement: S3 Fig — Data presented is for the COVID-19 related deaths in Pakistan between months April 2021 till February 2022. Source, John Hopkins, Corona Research Center https://coronavirus.jhu.edu/region/pakistan. (TIF) [file pgph.0001896.s003.tif]
