# Supplementary material for: Tracking SARS-CoV-2 variants through pandemic waves using RT-PCR testing in low-resource settings
Source: PLOS Glob Public Health. 2023 Jun 1;3(6):e0001896. doi: 10.1371/journal.pgph.0001896 (PMC10234525; doi:10.1371/journal.pgph.0001896)
Supplement: S1 Table — (DOCX) [file pgph.0001896.s004.docx]

**Supplementary Table 1.** **Timeline of SARS-CoV-2 variant testing kits employed**

| **Month Year** | **Predominant VOCs during monthly period** | **Kit A** | **Kit B** | **Kit C** | **Kit D** | **Kit E** | **Kit F** | **Target Spike mutations to identify VOC from PCR results** |
| --- | --- | --- | --- | --- | --- | --- | --- | --- |
| April 2021 | Alpha |  |  |  |  |  |  | Kit A: N501Y, A570D (Alpha); N501Y (Beta)  Kit B: N501Y (Alpha); N501Y, E484K, K417N (Beta); E484K, N501Y (Gamma) |
| May 2021 | Alpha |  |  |  |  |  |  | Kit B: N501Y (Alpha); N501Y, E484K, K417N (Beta); E484K, N501Y (Gamma)  Kit C: L452R (Delta) |
| June 2021 | Beta |  |  |  |  |  |  | Kit B: N501Y (Alpha); N501Y, E484K, K417N (Beta); E484K, N501Y (Gamma)  Kit C: L452R (Delta) |
| July 2021 | Delta |  |  |  |  |  |  | Kit B: N501Y (Alpha); N501Y, E484K, K417N (Beta); E484K, N501Y (Gamma)  Kit C: L452R (Delta)  Kit D: 681R (Delta) |
| August 2021 | Delta |  |  |  |  |  |  | Kit B: N501Y (Alpha); N501Y, E484K, K417N (Beta); E484K, N501Y (Gamma)  Kit D: 681R (Delta)  Kit E: E484, L452R (Delta/Epsilon); E484Q, L452R (Kappa); E484K (Gamma) |
| September 2021 | Delta |  |  |  |  |  |  | Kit B: N501Y (Alpha); N501Y, E484K, K417N (Beta); E484K, N501Y (Gamma)  Kit E: E484, L452R (Delta/Epsilon); E484Q, L452R (Kappa); E484K (Gamma) |
| October 2021 | Delta |  |  |  |  |  |  | Kit B: N501Y (Alpha); N501Y, E484K, K417N (Beta); E484K, N501Y (Gamma)  Kit E: E484, L452R (Delta/Epsilon); E484Q, L452R (Kappa); E484K (Gamma) |
| November 2021 | Delta |  |  |  |  |  |  | Kit B: N501Y (Alpha); N501Y, E484K, K417N (Beta); E484K, N501Y (Gamma)  Kit E: E484, L452R (Delta/Epsilon); E484Q, L452R (Kappa); E484K (Gamma) |
| December 2021 | Delta/Omicron |  |  |  |  |  |  | Kit B: N501Y (Alpha); N501Y, E484K, K417N (Beta); E484K, N501Y (Gamma)  Kit E: E484, L452R (Delta/Epsilon); E484Q, L452R (Kappa); E484K (Gamma)  Kit F: S-gene target failure, del 69–70 (Omicron) |
| January 2022 | Omicron |  |  |  |  |  |  | Kit F: S-gene target failure, del 69–70 (Omicron) |
| February 2022 | Omicron |  |  |  |  |  |  | Kit F: S-gene target failure, del 69–70 (Omicron) |

VOC, variant of concern. PCR Kit A: GSD Novatype SARS-CoV-2 , Eurofins. Kit B: GSD Novatype II SARS-CoV-2, Eurofins. Kit C: PhoenixDx SARS-CoV-2 Mutant Screen [L452R] by Promocure Biotech GmbH. Kit D: GSD NovaType Select P681R SARS-CoV-2, Eurofins. Kit E: GSD Novatype III SARS-CoV-2. Eurofins. Kit F: TaqPath™ COVID‑19 CE‑IVD RT‑PCR Kit, Applied Biosystems.

The shaded cells indicate the month/year when specific PCR kits were used. The table depicts the signature mutations used to identify VOC using the PCR kits.
