# Supplementary material for: Tracking SARS-CoV-2 variants through pandemic waves using RT-PCR testing in low-resource settings
Source: PLOS Glob Public Health. 2023 Jun 1;3(6):e0001896. doi: 10.1371/journal.pgph.0001896 (PMC10234525; doi:10.1371/journal.pgph.0001896)
Supplement: S2 Table — SARS-CoV-2 variants were identified with a PCR based approach targeting lineage specific mutations using commercially available assays. (A-F) present the specificity target of the probes/primers sequences and their target mutation of these assays as provided by the manufacturer. (DOCX) [file pgph.0001896.s005.docx]

**Supplementary Table 2. Details of SARS-CoV-2 variant testing PCR kits used**

**Kit A. GSD NovaType SARS-CoV-2, Eurofins**

| Fluorescent Channels | | Results and Interpretation |
| --- | --- | --- |
| HEX/VIC | FAM |  |
| + | - | SARS-CoV-2 Wild type detected |
| - | + | N501Y,A570D - Alpha variant detected |
| + | + | N501Y - Beta variant detected |

Kit limit:

- Positive: Ct ≤ 38
- Negative (neg): Not detected (ND) or Ct > 38

**Kit B. GSD NovaType II SARS-CoV-2, Eurofins**

| Fluorescent Channels | | | | Results and Interpretation |
| --- | --- | --- | --- | --- |
| HEX/VIC | CY5 | FAM | ATTO425 |  |
| + | - | - | + | N501, RNAse P - Wild type SARS-CoV-2 detected |
| - | - | + | + | N501Y, RNAse P - Alpha variant detected |
| + | + | + | + | N501Y, E484K, K417N, RNAse P Beta variant detected |
| - | + | + | + | E484K, N501Y, RNAse P - Gamma variant detected |
| - | - | - | + | RNAse P - Negative - SARS-CoV-2 not detected |

Kit limit:

- Positive: Ct ≤ 38
- Negative (neg): Not detected (ND) or Ct > 38

**Kit C. PhoenixDx SARS-CoV-2 Mutant Screen [L452R], Promocure Biotech GmbH**

| Fluorescent Channels | | Results and Interpretation |
| --- | --- | --- |
| FAM | HEX/VIC |  |
| - | - | Negative - SARS-CoV-2 not detected |
| + | - | 452L - Wild type SARS-CoV-2 detected |
| + | + | L452R - Delta variant detected |

Kit limit:

- Positive: Ct ≤ 35
- Negative (neg): Not detected (ND) or Ct > 35

**Kit D. GSD NovaType Select P681R SARS-CoV-2, Eurofins**

| Fluorescent Channels | | | Results and Interpretation |
| --- | --- | --- | --- |
| FAM | HEX/VIC | CY5 |  |
| - | - | + | RNAse P - Negative SARS-CoV-2 not detected |
| - | + | + | P681 - Wild type SARS-CoV-2 detected |
| + | - | + | 681R, RNAse P - Delta variant detected |

Kit limit:

- Positive: Ct ≤ 38
- Negative (neg): Not detected (ND) or Ct > 38

**Kit E. GSD NovaType III SARS-CoV-2, Eurofins**

| Fluorescent Channels | | | | | Results and Interpretation |
| --- | --- | --- | --- | --- | --- |
| ROX | HEX/VIC | CY5 | FAM | ATTO425 |  |
| - | + | - | - | + | E484, RNAse P - Wild type SARS-CoV-2 detected |
| + | + | - | - | + | E484, L452R, RNAse P - Delta/Epsilon variant detected |
| + | - | - | + | + | E484Q, L452R, RNAse P – Kappa variant detected |
| - | - | + | - | + | E484K, RNAse P - Beta/Gamma variant detected |
| - | - | - | - | + | RNAse P - Negative - SARS-CoV-2 not detected |

Kit limit:

- Positive: Ct ≤ 38
- Negative (neg): Not detected (ND) or Ct > 38

**Kit F. TaqPath™ COVID‑19 CE‑IVD RT‑PCR Kit, Applied Biosystems**

| Fluorescent Channels | | | | Results and Interpretation |
| --- | --- | --- | --- | --- |
| HEX/VIC | ABY | FAM | JUN |  |
| + | + | + | + | ORF1ab, N, S and MS2 control - SARS-CoV-2 detected |
| + | - | + | + | ORF1ab, N and MS2 control - SARS-CoV-2 detected.  S-gene target failure (SGTF) suspected Alpha/Omicron |
| - | - | - | + | MS2 control (Negative - SARS-CoV-2 not detected) |

Kit limit:

- Positive: Ct ≤ 37
- Negative (neg): Not detected (ND) or Ct > 37

The above details for Kits A - F identify the target mutations and probes used to identify SARS-CoV-2 VOC in each case
