# Supplementary material for: Tracking SARS-CoV-2 variants through pandemic waves using RT-PCR testing in low-resource settings
Source: PLOS Glob Public Health. 2023 Jun 1;3(6):e0001896. doi: 10.1371/journal.pgph.0001896 (PMC10234525; doi:10.1371/journal.pgph.0001896)
Supplement: Attachment — Submitted filename: Reviewers comments 06.04.2023-zh.docx [file pgph.0001896.s007.docx]

Reviewer #1: 

*1. Are there controversies in this field? What are the most recent and important achievements in the field? In my opinion, answers to these questions should be emphasized. Perhaps, in some cases, novelty of the recent achievements should be highlighted by indicating the year of publication in the text of the manuscript.*

-We have discussed the importance of the expansion of genomic sequencing and surveillance efforts through the COVID-19 pandemic. We have also discussed in greater detail the pros and cons of using WGS and the use of RT-PCR based assays for surveillance.

The data described here contributed towards the National Institute of Health (Islamabad) SARS-CoV-2 VOC situational reports and influence policy making for the control of the virus in Pakistan. This achievement is mentioned in the discussion. The documentation of the increased transmissibility of the VOC has potential implications for hospital and health system capacity and control measures.

*2. The results and discussion section is very weak and no emphasis is given on the discussion of the results like why certain effects are coming in to existence and what could be the possible reason behind them?*

-We have re-written the results and discussion. An additional two figures (2A and 3) have been included to examine the results further. The discussion is written in much greater depth.

*3. Conclusion: not properly written.*

- We have revised the conclusions.

*4. Results and conclusion: The section devoted to the explanation of the results suffers from the same problems revealed so far. Your storyline in the results section (and conclusion) is hard to follow. Moreover, the conclusions reached are really far from what one can infer from the empirical results.*

*-*We have revised the results and conclusion. Repetition has been removed and the storyline is written more clearly.

*5. The discussion should be rather organized around arguments avoiding simply describing details without providing much meaning. A real discussion should also link the findings of the study to theory and/or literature.*

The results have been discussed with inclusion of supporting literature. Conclusion has been re-written highlighting the contributions of this study in a national effort of VOC surveillance.

*6. Spacing, punctuation marks, grammar, and spelling errors should be reviewed thoroughly. I found so many typos throughout the manuscript.*

*­-*Grammatical mistakes have been corrected and spellings have been reviewed throughout the manuscript.

*7. English is modest. Therefore, the authors need to improve their writing style. In addition, the whole manuscript needs to be checked by native English speakers.*

- Thank you. The manuscript has been checked by an native English speaker.

Reviewer #2:

*1. Results section, in screening for predominant VOCs there is some repetition of statements from the methodology, information such as primers, and mutations are already mentioned in the methods section. It is, therefore, better to remove the subsection to avoid redundancy.*

Thank you for pointing this out. We have modified this section to remove redundancies. The technical description of the kits is removed and we focus here on the rationale for the sequential usage of the RT-PCR assays.

*2. Supplementary Table 3 is not traceable in the manuscript file.*

Sorry that this attachment was missed earlier. Supplementary Table 3 is provided with the revised document.

*3. Authors should show or provide the VOCs detection concordance % between the specific-mutation PCR technique versus NGS. Alternatively, provide sensitivity and specificity for the PCR- a technique in the detection of VOCs.\*

We have provided Supplementary Table 3 which shows the concordance between VOCs detected by the specific mutation PCR and NGS methods for 49 samples. This was available for a limited number of samples therefore, we are unable to provide complete sensitivity and specificity information for the full data set, this has also been mentioned in the study limitations.
Further, we have explained the inclusion criteria for the samples we selected for RT-PCR testing, explaining that these were samples up to a CT of 35.

Reviewer #3:

*1. The authors reported several kits employed for identifying various VOCs over time, but never mention previous literature (such as PMID: 35196812, PMID: 35204558, PMID: 35367360). On this subject, the discussion of studies with similar approach could also help for highlighting novel elements in the present studies. This part should be mentioned in the Introduction and then illustrated in the Discussion.*

Thank you for pointing this out. We have cited the recommended articles and also referred to them in the Discussion.

*2. Furthermore, the manuscript does not fully describe the differences between the identified VOCs and more details on how each kit is able to identify the variants are needed. To this end, a suggestion would be to add a column to Supplementary Table 1 with the mutations related to each VOC.*

The suggestion made has been incorporated into our supplementary materials. Supplementary Table 1 has an additional column listing the mutations identified by each kit that were used to identify each VOC. Also, an additional figure (Supplementary Figure 1) has been included which depicts the workflow of how each RT-PCR kit was utilized to screen and differentiate the VOC.

*3. Did the authors perform an evaluation of the limit of detection (in terms of viral load) for the variants during the related wave?*

The SARS-CoV-2 cobas 6800 Roche diagnostic assay we used identifies positive samples at a 38 Ct value cut off (100 cp/ml as per the manufacturer’s recommendation and validated by our laboratory as per College of American Pathologists guidelines). We selected samples with a CT value of 35 or below for specific mutation PCR testing.

Due to shortage of reagent kits, we did not conduct a full LOD analysis. Although we did not conduct a comprehensive limit of detection testing for each kit, we observed that for samples with CT >35, the mutation specific PCR test results were not consistent. We can say therefore state that the mutation PCR approach is not recommended for lower viral load samples. *This point has now been included in the limitations.*

*4. The authors should dwell more on the analysis and methods. More specifically, they should better describe the methods and the investigation that led them to make a correlation between VOC and COVID-19 severity in their case study. They should describe the employed statistical approach.*

We have described our methods and analysis further. As stated in the Methods section, for specific mutation PCR testing, we selected samples with a CT value of 35 or below. As we have now included an additional figure to address the point raised. Figure 3 shows the viral loads (CT) values of the VOC. A statistical analysis is included in the description of Figure 3.*.*

*5. In the text, the authors very often mention PCR instead of RT-PCR. I suggest using only RT-PCR for the sake of clarity.*

PCR has been replaced by RT-PCR throughout the manuscript.

*6. I could not find the Supplementary table 3. On this subject, I reckon that in lines 238-241 the precise number of samples tested both by RT-PCR and WGS should be mentioned to make the study more reliable.*

Thank you for pointing this out. Supplementary Table 3 was missed. It contains information for WGS of 49 samples whereby VOC were also identified by RT-PCR. This table has now been provided with the revised manuscript.
